# Contralateral suppression of transient evoked otoacoustic emissions in adults: A normative study

**DOI:** 10.4102/sajcd.v69i1.929

**Published:** 2022-12-08

**Authors:** Simone Zevenster, Alida Naudé

**Affiliations:** 1 Department of Audiology, School of Advanced Education, Research and Accreditation, Castellón de la Plana, Spain; 2 Centre for Augmentative and Alternative Communication, Faculty of Humanities, University of Pretoria, Pretoria, South Africa

**Keywords:** contralateral suppression, transient evoked otoacoustic emission, normative data, otoacoustic emissions, efferent system, medial olivocochlear bundle

## Abstract

**Background:**

Whilst otoacoustic emission (OAE) testing has proved to be valuable in revealing information about cochlear outer hair cell integrity, it does not provide insight into the afferent and efferent pathways once the stimulus has reached neural receptors. This information can be obtained objectively through contralateral acoustic stimulation (CAS) suppression. However, obtaining normative data is essential in the implementation of such tests.

**Objectives:**

The primary aim was to undertake a small pilot study to collect the CAS suppression across a predefined frequency range in order to provide a preliminary normative data set to be used with the newly developed transient evoked otoacoustic emission (TEOAE)-CAS module (PATH MEDICAL, Germering, Germany). Secondary aims included the analysis of the relationships between left and right CAS suppression, between male and female CAS suppression and between TEOAE signal-to-noise ratio (SNR) and CAS suppression.

**Methods:**

The purpose of this study was to determine preliminary normative data for contralateral TEOAE suppression from 40 normal ears of 20 healthy young adults (10 males and 10 females). Subjects were recruited using purposive sampling. The CAS suppression responses were obtained automatically by means of the data-collection protocol on the device used. From the data obtained, correlations between TEOAE SNR and CAS suppression were made using Pearson’s correlation coefficient.

**Results:**

The data were statistically processed to form a normative database which possesses the potential of serving as a basis for further research aimed at determining the utility of CAS suppression testing when evaluating ear pathology. A mean CAS suppression of 0.8 decibels (dB) (0.61 SD) was obtained. There was no statistically significant relationship between TEOAE SNR and CAS suppression. There was no significant suppression difference in terms of laterality of ears or gender.

**Conclusion:**

Normative values for CAS suppression of TEOAEs in a group of normal-hearing individuals were obtained using the newly developed TEOAE-CAS module (PATH MEDICAL, Germering, Germany). The availability of normative data for contralateral TEOAE suppression using the studied module allows for it to become commercially available, which will enable researchers and audiologists to perform this measurement in different populations in the evaluation of ear pathology.

## Introduction

Early pioneering research led to two audiological discoveries which have initiated a greater understanding and prompted further studies of how the auditory system functions. The first is that of the efferent auditory pathway between the cochlea and the brain (Rasmussen, 1946). Years later, the discovery of the otoacoustic emission (OAE) by David Kemp allowed clinicians to obtain a better understanding of cochlear functioning (Kemp, Bray, Alexander, & Brown, 1986). The coalescence of these findings, combined with recent hearing research, has resulted in further understanding of how the peripheral and central hearing systems function together.

Otoacoustic emissions are generally understood to be acoustic sounds that originate secondary to movement of outer hair cells (OHCs) (Robinette, Cevette, & Probst, 2007). Unlike other sensory receptor systems, the cochlea generates signals of the same type as that which it is designed to receive (Siegel, 2008). During the process of sensory transmission and transduction, both an afferent signal to the auditory nerve and an efferent signal travelling back through the middle ear to the outer ear canal are elicited (Siegel, 2008). It is here that an OAE can be measured (McFadden, Pasanen, Raper, Lange, & Wallen, 2006). A present OAE suggests normal pre-neural OHC functioning and middle-ear status (Nobili, Vetešnik., Turicchia, & Mammano, 2003). Not only can an OAE test be used to objectively determine whether the cochlea is responding to sound, but studies are showing that an OAE response can also change with sound presented to the opposite ear (Garinis, Glattke, & Cone-Wesson, 2008; Geven, De Kleine, Free, & Van Dijk, 2011). Contralateral noise presented during a recording has been shown to result in a reduction of the OAE amplitude in normal-hearing individuals, also referred to as the suppression of OAEs (Joseph, Suman, Jayasree, & Prabhu, 2019).

There are several types of OAEs, namely spontaneous, distortion product and transient evoked OAEs (TEOAEs). Transient evoked OAEs, the focus of this study, are evoked by a brief sound stimulus (usually a click) that has a wide (broadband) range of frequencies (Vasconcelos, Serra, & De Farias Aragão, 2008). Using a nonlinear click train protocol ensures that the stimulus artefacts of a linear nature are removed from the recording, allowing for maximum reliability of the TEOAE recording (Hatzopoulos, Petrucell, Morkt, & Martini, 2003; Von Specht, Ganz, Pethe, Leuschner, & Pytel, 2001). This article specifically focuses on the amount of contralateral TEOAE suppression, from here on referred to as contralateral acoustic stimulation (CAS) suppression, that is found in a group of normal-hearing adults.

Whilst OAE testing has proved to be valuable in revealing information about OHC integrity, it does not provide insight into the afferent and efferent pathways once the stimulus has reached neural receptors. The effect of contralateral noise on OAE amplitude has been reported to be caused by the reduction in gain from the cochlear amplifier which is modulated by efferent pathways (Guinan, 2006). Efferent pathways originate from the superior olivary complex (SOC), and these descending fibre bundles provide direct, bilateral input to the cochlea via two efferent divisions, namely lateral and medial (Kumar, Grover, Publius, Sanju, & Sinha, 2016). The medial efferent division is known as the medial olivocochlear bundle (MOCB) and innervates and modulates the OHCs of the cochlea (Kumar et al., 2016). It is therefore assumed that stimulation of the MOCB will result in an alteration of OHC motility and hence affect OAEs. The main effect of contralateral stimulation on TEOAEs is the reduction of ipsilateral TEOAE amplitude of about 1 dB – 4 dB (Berlin et al., 1993; Berlin, Hood, Hurley, & Wen, 1994; Collet et al., 1990; Collet, Veuillet, Bene, & Morgan, 1992; Ryan, Kemp, & Hinchcliffe, 1991; Veuillet, Collet, & Duclaux, 1991). Therefore, the TEOAE amplitude is thought to be suppressed by contralateral stimulation by up to 4 dB (Berlin et al., 1993, 1994; Collet et al., 1990, 1992; Ryan et al., 1991; Veuillet et al., 1991).

The amount of suppression (CAS response) is dependent on the type of stimulus used. White noise, which consists of energy from 20 Hz to 20 000 Hz, stimulates the whole contralateral cochlear portion and activates the largest number of MOCB efferents, thus making it an effective stimulus in suppressing TEOAEs (Kalaiah, Nanchirakal, Kharmawphlang, & Noronah, 2017). Further stimulus parameters can change the CAS suppression. In a study conducted on the appropriate click and noise levels for testing CAS suppression, it was found that lower OAE intensity level clicks yielded greater amplitude suppression when the intensity of the contralateral noise was approximately 60 dB sound pressure level (SPL) (Hood, Berlin, Hurley, Cecola, & Bell, 1996a). Furthermore, Hood et al. (1996a) suggested using 55 dB or 60 dB peak equivalent (p.e.) SPL for ipsilateral OAE testing with the overall intensity level of the contralateral noise set at, or 5 dB higher than, the ipsilateral OAE click intensity. It is important to avoid using higher OAE click intensities, such as 70 dB SPL, to reduce the risk of middle-ear muscle reflex participation (acoustic reflex) (Guinan, 2006; Velenovsky & Glattke, 2002).

Contralateral acoustic stimulation suppression testing is not yet routinely performed in clinical practice and not considered part of the basic test battery (Hall, 2017). The reason for this might be because acoustic reflex threshold (ART) testing is used in the test battery to assess for the site of lesion of the efferent pathway (Kung & Willcox, 2007). However, more information about the functioning of the MOCB can be obtained from the presence, absence and amount of suppression (Muchnik et al., 2004).

Although limited, research holds promise for the usefulness of CAS suppression tests in the diagnosis of pontine lesions, either extrinsic (e.g. acoustic neuromas) or intrinsic (e.g. multiple sclerosis) (Prasher, Ryan, & Luxon 1994). Measures of CAS suppression can therefore indicate the status of the functioning of these olivocochlear bundle (OCB) efferent nerve fibres (Kumar et al., 2016). Furthermore, CAS suppression can provide information about the interaction between afferent and efferent pathways and the interaction between peripheral and central hearing loss, thereby aiding in differential diagnosis (Hood, Berlin, Hurley, & Wen, 1996b). In a study done by Hood et al. (1996b), it was recommended that TEOAE suppression be used in patients with auditory neuropathy as a differential measure of auditory function.

The importance of measures that are efficient and effective in forming part of the audiological test battery is well known. Hall (2017) discusses the importance of using tests that are not only sensitive but also specific. One aspect of this speaks to the reliability of a measure. In a study done on the reliability of CAS suppression, it was found that the suppression measures were reliable across test sessions of 1–2 days, and it concluded that CAS suppression provides a good test of the MOCB system over time (Stuart & Cobb, 2015).

Normative data on CAS suppression in literature are limited. Norms for the manual recording of TEOAEs before and after contralateral stimulation have been done using middle-ear analysers from Grason-Stadler and Otodynamics to record the TEOAE and audiometers from Natus and Grason-Stadler to present the contralateral noise (Garinis et al., 2008; Geven et al., 2011; Van Zyl, Swanepoel, & Hall, 2009). One study used the ILO292 from Otodynamics to record the TEOAE response in the ipsilateral ear with probe 1 and presented the contralateral white noise with probe 2 (Abdollahi & Lotfi, 2011). However, the device did not calculate the suppression automatically, and the researchers were required to perform manual calculations.

The primary aim of the current study was to undertake a small pilot study to collect the CAS suppression across a predefined frequency range in order to provide an appropriate normative data set to be used with the newly developed TEOAE-CAS module (PATH MEDICAL, Germering, Germany) which is not yet commercially available, making this the first study on this specific module. One must consider that each device used for audiological assessment (and the protocols selected within that device), each clinical test environment and each patient is unique. Therefore, it is particularly important to have normative data gathered from the specific device used (Campbell, 2013). Secondary aims of the current study included the analysis of the relationships between left and right CAS suppression, between male and female CAS suppression and between TEOAE signal-to-noise ratio (SNR) and CAS suppression.

Normative data would allow researchers to gain a better understanding of the relationships between the efferent pathway and other audiological tests such as ART, pure tone audiometry (PTA) and speech audiometry testing. Furthermore, it will provide the opportunity to compare results with other tests of auditory nerve functioning, such as auditory evoked potentials. Establishing norms allows for further research and increases the potential for CAS suppression to be used as a clinical audiological investigation tool in the evaluation of tinnitus, learning difficulties, acoustic trauma and hyperacusis (Garinis et al., 2008; Urnau & Tochetto, 2012).

## Methods

A nonexperimental analytical quantitative research design was used to obtain normative data, because this design does not require the manipulation of an independent variable (Edmonds & Kennedy, 2017). Normative data describes values that are usual in a reference group and allows for comparison to subsequent values (Campbell, 2013).

### Subjects

Purposive sampling was used to obtain subjects with normal bilateral auditory sensitivity. Normal auditory sensitivity was defined as air conduction PTA ≤ 20 dB hearing level (HL) for test frequencies at octave intervals from 1000 Hz to 4000 Hz. Normal middle-ear function indicated by type A tympanograms and present ipsilateral and contralateral acoustic reflexes were required. All subjects included in the study had no noticeable abnormalities according to the otoscopic examination, present and normal TEOAE responses at 80 dB p.e. SPL bilaterally and no history of ototoxic drug treatment, chronic middle-ear disease, or family history of hereditary deafness. Data were obtained from 40 ears, 10 females and 10 males, with an age range of 18–25 years. A summary of subjects’ ages is shown in Table 1. Written consent was obtained from each subject.

**TABLE 1:** Summary of subjects’ ages.

Variables	Group	Female	Male
Mean	21.00	20.70	21.20
Standard deviation (SD)	2.31	2.50	2.20
Median	20.50	20.50	20.50
Range	18–25	18–25	18–24

### Data collection

#### General protocol

**FIGURE 1:**
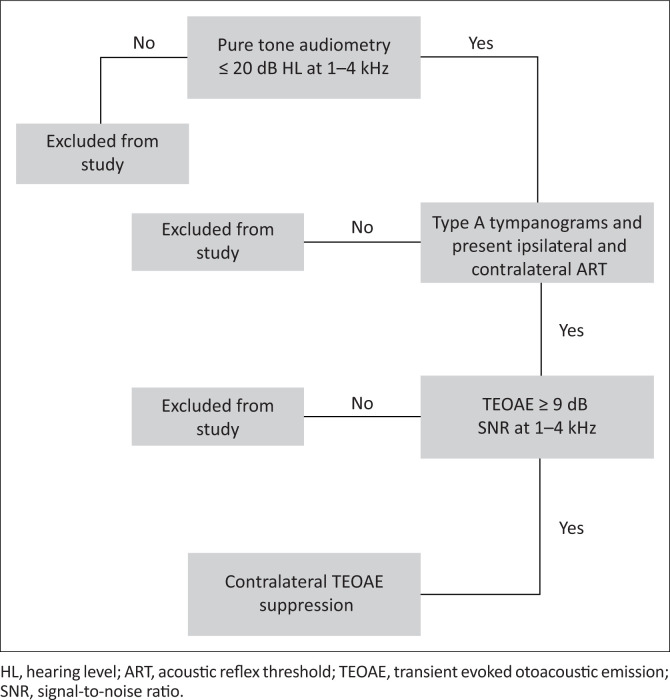
Protocol for testing whether subjects met the selection criteria.

The general protocol included case history as well as audiometric, immittance and TEOAE testing in a sound-insulated booth to identify those participants complying with the inclusion criteria. Continuation of the protocol was dependent on the results of the tests noted above. Figure 1 denotes the protocol used to determine if participants met the selection criteria.

All 40 ears were required to present with normal auditory sensitivity and middle ear functioning as summarised in Table 2. When normal auditory sensitivity was indicated, CAS suppression was recorded by means of two OAE probes: one to record the response in the ipsilateral ear and the other to present the contralateral stimulus.

**TABLE 2:** Results of acoustic reflex threshold and pure tone audiometry from normal ears (*n* = 40).

Variable	ART (dB HL)				PTA (dB HL) right	PTA (dB HL) left
	Ipsilateral		Contralateral			
	Right	Left	Right	Left		
Subject 1	75	80	70	95	0	−3
Subject 2	70	85	100	85	4	5
Subject 3	90	85	90	85	−7	−4
Subject 4	90	85	90	100	0	1
Subject 5	90	85	85	85	8	4
Subject 6	85	85	85	85	−5	−1
Subject 7	70	70	85	80	−3	−6
Subject 8	90	85	95	80	−4	−2
Subject 9	75	80	70	80	6	−3
Subject 10	75	80	80	90	−6	−2
Subject 11	75	80	90	85	11	4
Subject 12	90	95	95	95	2	−2
Subject 13	85	80	95	80	0	3
Subject 14	90	85	100	100	6	9
Subject 15	85	90	85	90	5	0
Subject 16	85	90	85	90	7	6
Subject 17	75	100	80	85	1	5
Subject 18	80	95	85	85	6	6
Subject 19	70	95	100	95	8	4
Subject 20	90	90	85	95	−3	−3
Mean	82	86	88	88	2	1

ART, acoustic reflex threshold PTA, pure tone audiometry; HL, hearing level.

#### Transient evoked otoacoustic emission testing

The Sentiero Advanced TEOAE module (PATH MEDICAL, Germering, Germany) was used to record TEOAE responses using an 80 dB p.e. SPL nonlinear click train. A TEOAE was considered present and normal if a response spectral peak was apparent at 9 dB above the noise floor in all third-octave bands from 1000 to 4000 Hz (Mishra & Lutman, 2013; Stuart & Cobb, 2015).

#### Contralateral acoustic stimulation suppression responses

**FIGURE 2:**
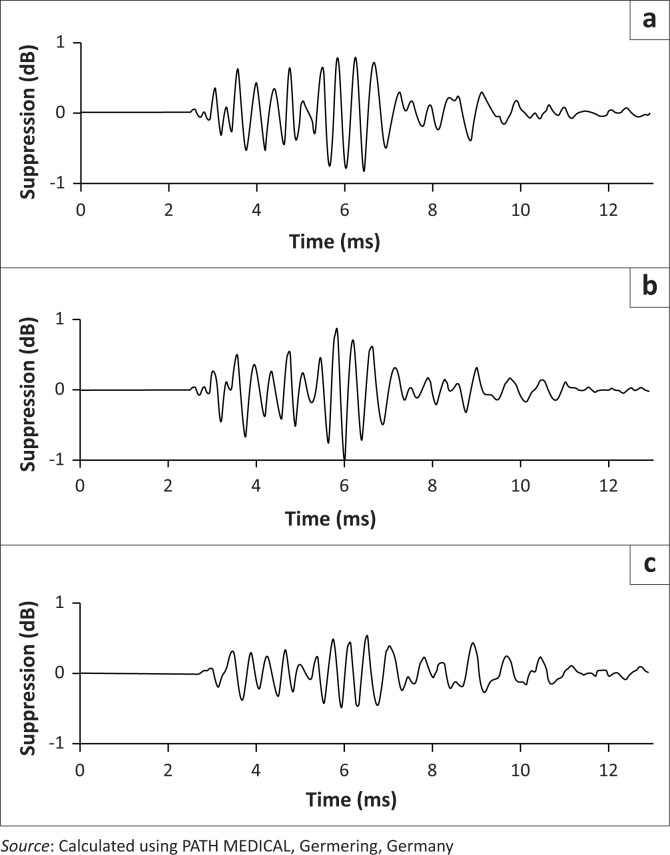
Graphical representation of the suppression calculation. (a) Unsuppressed, (b) suppressed and (c) difference.

The Sentiero Advanced TEOAE-CAS module (PATH MEDICAL, Germering, Germany) was used to measure CAS suppression responses. Contrary to other manufacturers, this module comprises a proprietary algorithm which uses the TEOAE click stimulus rather than white noise as the contralateral suppressor stimulus for the other ear. The method employs a stimulus sequence that records TEOAE unsuppressed and TEOAE suppressed for both ears simultaneously by alternating between ears. The method uses two averaging buffers per ear, collecting unsuppressed and suppressed TEOAE separately. Suppression effect was detected by calculating the difference trace between these buffers and performing a signal statistical evaluation on the difference trace, which is done automatically on the device. Figure 2 shows a graphical representation of the calculation of the suppression effect. Two consecutive trials of the CAS suppression recordings were done, with subjects instructed to remain quiet and motionless to avoid displacement of the probe tip.

#### Statistical analysis

The data were analysed quantitatively and captured electronically and were analysed using the data analysis function in Microsoft Excel 365, version 2104. Additionally, descriptive statistics were employed to describe and summarise the data. The data were described in terms of the mean CAS suppression and standard deviation and further correlated with TEOAE SNR. The data were then correlated for gender and laterality differences, using Pearson’s correlation coefficient.

### Ethical considerations

Ethics approval was obtained from the Research Ethics Committee of the University of Pretoria, South Africa, (reference number: 04348664 [HUM030/0519]).

## Results

### Test-retest reliability of contralateral acoustic stimulation suppression

Test-retest reliability of CAS suppression within the same session was analysed by computing the correlation between the CAS suppression responses from the first and second measurement using Pearson’s correlation coefficient. Test-retest reliability was confirmed by a strong correlation for CAS suppression, *r* = 0.806 (*p* = < 0.001) in conditions of not more than 5 min between recordings. Van Zyl et al. (2009) also showed that a stable TEOAE suppressive effect over 5-min intervals was obtainable. This means that the MOCB can recover from a suppression effect on OHCs within 5 min.

### Transient evoked otoacoustic emission signal-to-noise ratio

All 40 ears showed clear TEOAE responses for SNR of ≥ 9 dB at 1000 Hz – 4000 Hz bilaterally using an 80 dB p.e. SPL nonlinear click stimulus. Table 3 shows the data obtained for TEOAE SNR for each frequency. The TEOAE total overall SNR ranged between 9 dB and 29.7 dB with a mean of 15.91 dB (SD 5.53). Although the TEOAE SNR for right ears seemed lower than that of left ears, there was no statistically significant difference (*p* = > 0.05).

**TABLE 3:** Right and left transient evoked otoacoustic emission’s signal-to-noise ratio (dB) from normal ears (*n* = 40).

Frequency (Hz)	Right ear		Left ear	
	SNR	SD	SNR	SD
1000	12.44	3.50	13.00	4.49
1500	17.55	5.14	18.74	5.53
2000	18.97	4.38	19.69	4.75
3000	18.12	3.85	19.17	4.86
4000	10.27	2.85	11.17	3.38
*p* value	-	0.73	-	-

SD, standard deviation; SNR, signal-to-noise ratio.

### Contralateral transient evoked otoacoustic emission’s suppression

The mean CAS suppression responses were determined with the main aim of the study in mind. Table 4 shows the data collected for CAS suppression bilaterally. The mean CAS suppression determined for this study sample was 0.8 dB. The data were calculated into percentiles, which are depicted in Table 5. Data indicated suppression of 1.1 dB (for example) in the 70th percentile. This means that 70% of suppression values from the sample were 1.1 dB or lower. A CAS suppression value of 0.5 dB was found at the 30th percentile. Therefore, 30% of the suppression values were 0.5 dB or lower, and 70% were higher than 0.5 dB. Thus, the mean CAS suppression obtained in this study falls between the 50th and 60th percentile. The suppression for the lower limit (5th percentile) was 0.0 dB, and the suppression for the upper limit (95th percentile) was 1.8 dB.

**TABLE 4:** Contralateral acoustic stimulation suppression (dB) from normal ears (*n* = 40).

Variable	Value
Mean	0.8 dB
Standard deviation (SD)	0.61
Mean −1 SD	0.19
Mean −2 SD	−0.42
Mode	0.70
Median	0.70
Range min (dB) – max (dB)	−0.1 to 2.5

### Comparison between transient evoked otoacoustic emission’s signal-to-noise ratio and contralateral acoustic stimulation suppression

**FIGURE 3:**
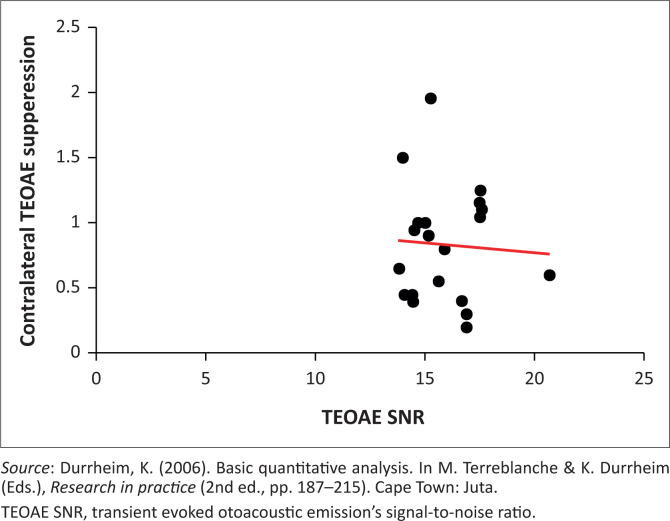
Linear regression graph of transient evoked otoacoustic emission’s signal-to-noise ratio and contralateral acoustic stimulation suppression.

To determine the degree to which suppression was a function of TEOAE SNR, TEOAE SNR and CAS suppression were correlated using Pearson’s correlation coefficient. As indicated by the *p* value, no statistically significant relationship, *r*(18) = 0.06, *p* = > *α* (*α* = 0.05) was found between TEOAE SNR and CAS suppression. These results are represented graphically in Figure 3. The correlation coefficient depicted by a scatterplot cluster around the regression line confirms that these two variables are not related to one another.

### Contralateral acoustic stimulation responses in right and left ears

Contralateral acoustic stimulation suppression for the right and left ears are summarised in Table 6. Using a two-sample *t*-test, the difference between mean right and left values was not statistically significant (*p* = > 0.05), suggesting that the efferent pathways reacted similarly to sound presented bilaterally in this group of subjects.

**TABLE 5:** Normative data for the percentile values of suppression level for a transient evoked otoacoustic emission’s click.

Percentile (%)	Suppression level dB
1	−0.1
5	0.0
10	0.1
20	0.4
30	0.5
40	0.7
50	0.7
60	0.9
70	1.1
80	1.3
90	1.7
95	1.8
99	2.3

**TABLE 6:** Contralateral acoustic stimulation suppression for right and left ears (*n* = 40).

Variable	Right ear	Left ear
Mean	0.8800	0.7900
Variance	0.3283	0.4241
Observations	20.0000	20.0000

Note: Pooled variance = 0.3762; hypothesised mean difference = 0.0000; degrees of freedom = 38.0000; *T* stat = 0.4382; *P*(*t* ≤ *t*) one-tail = 0.3318; *T* critical one-tail = 1.6860.

### Contralateral acoustic stimulation suppression in women and men

Mean female and male CAS suppression were analysed using a two-sample *t*-test assuming equal variance. Contralateral acoustic stimulation suppression for female and male subjects are summarised in Table 7. The gender differences were shown not to be statistically significant (*p* = > 0.05), suggesting that normative data obtained could be used across both genders.

**TABLE 7:** Contralateral transient evoked otoacoustic emission’s suppression data for females and males (*n* = 40).

Variable	Females	Males
Mean	0.8400	0.8000
Variance	0.1824	0.3020
Observations	10.0000	10.0000

Note: Pooled variance = 0.2422; hypothesised mean difference = 0.0000; degrees of freedom = 18.0000; *T* stat = 0.1727; *P*(*t* ≤ *t*) one-tail = 0.4324; *T* critical one-tail = 1.7341.

## Discussion

This study was primarily intended to determine the CAS suppression elicited in a group of normal-hearing young adults to construct normative data. Analysis of the results showed clearly measurable suppression for all 40 ears. This study correlated with other studies that showed the suppression of TEOAEs using a nonlinear click train with the introduction of a contralateral TEOAE stimulus (Abdollahi & Lotfi, 2011; De Ceulaer et al., 2001; Garinis et al., 2008; Geven et al., 2011).

Results indicated a range of −0.1 dB (minimum suppression value) and 2.5 dB (maximum suppression value), with a mean value of 0.8 dB. One-third of suppression values were 0.5 dB or less, which implies that 70% of the suppression values obtained were higher than 0.5 dB. This finding correlates with a previous study reporting normal suppression values to be 0.5 dB – 1 dB (Collet et al., 1992). This agreement suggests that automated calculation of CAS suppression is a valuable addition to the test battery, increasing the efficacy of the procedure. A study performed by De Ceulaer et al. (2001) reported normative suppression values as low as −0.35 dB and as high as 3.89 dB. They found that optimal suppression was obtained using a click level of 12 dB above each individual’s TEOAE response threshold and a contralateral white noise at 40 dB SL. These minor differences emphasise that normative values from each study should be evaluated in accordance with the specific stimulus parameters employed in the study and that different equipment used to measure CAS suppression may result in different normative data. This information highlights the importance of the aim of this study to obtain equipment-specific normative data.

There was no statistically significant relationship between TEOAE SNR and CAS suppression. For the use as a differential test, these results are expected to provide insight into how CAS suppression tests could be used to identify cochlear dysfunction in patients with normal-hearing sensitivity and suppression but with abnormal TEOAE responses or, conversely, identifying MOCB dysfunction by the presence of TEOAEs and absence of suppression. Such a study was conducted by Sarathy and Jaya (2017), where subjects with tinnitus and normal hearing sensitivity were evaluated in terms of TEOAE responses and CAS suppression. The findings from this study showed that abnormal TEOAE in patients with tinnitus having normal hearing sensitivity indicated cochlear dysfunction, with the absence of suppression indicating MOCB dysfunction (Sarathy & Jaya, 2017).

Findings related to TEOAE SNR and CAS suppression of the left and right ears agree with other studies of TEOAE responses, suppression and laterality (Muchnik et al., 2004; Sanches & Carvallo, 2006). However, asymmetries in auditory function have been reported at different levels of the auditory nervous system (Garinis et al., 2008). In the study by Garinis et al. (2008), a notable difference in the amount of suppression occurred in the right-ear control group (normal hearing). A significant difference in laterality would indicate the need for separate normative data for left and right ears. The current study, however, found no statistically significant difference between left and right ears and therefore the normative range could be applied binaurally.

Similarly, no statistically significant gender difference was observed related to CAS suppression. Other studies have, however, reported gender differences (Abdollahi & Lotfi, 2011; Cassidy & Ditty, 2001). Mean suppression values from 60 normal-hearing participants, using a similar parameter setup to that of the current study, were found to be 2.07 dB in males and 1.54 dB in females, which was statistically significant (Abdollahi & Lotfi, 2011). Durante and Carvallo (2006) also found larger suppression values in neonate males than females. One potential reason for the current finding being in contrast with these studies might be related to their larger sample sizes. Researchers do not know the exact reason for gender differences in suppression, but there are some hormonal and structural explanations for these findings (Abdollahi & Lotfi, 2011).

Research has shown that larger suppression amplitude for emissions can be obtained with clicks with a lower intensity level when the intensity of the contralateral noise was at or near 60 dB SPL (Hood et al., 1996a). This study, however, was in accordance with the results from Hood et al. (1996b) stating that contralateral intensity levels set at, or 5 dB higher than, the ipsilateral click intensity, indicated positive CAS suppression. Therefore, this data can be used for further studies on the TEOAE-CAS module using differing stimulus parameters.

## Conclusion

Contralateral acoustic stimulation suppression is a relatively underused area of research in the field of audiology, with a potential future in applications of objective diagnosis of pathologies of the MOCB. Having normative data on CAS suppression that are specific to a device to maximally interpret the data acquired on an individual can allow for further research on pathologies of the efferent system. Observations on suppression in patients with tinnitus, auditory processing, hyperacusis and auditory neuropathy, as well as other intrinsic or extrinsic lesions, can be clinically and objectively evaluated and interpreted using specific normative data obtained on a larger group of subjects. Furthermore, the evaluation of CAS suppression can also be further studied with regard to differences between apparatus used.

## References

[CIT0001] Abdollahi, F.Z., & Lotfi, Y. (2011). Gender difference in TEOAEs and contralateral suppression of TEOAEs in normal hearing adults. *Iranian Rehabilitation Journal*, 9(14), 22–25.

[CIT0002] Berlin, C.I., Hood, L.J., Hurley, A., & Wen, H. (1994). Contralateral suppression of otoacoustic emissions: An index of the function of the medial olivocochlear system. *Otolaryngology – Head and Neck Surgery*, 110(1), 3–21. 10.1177/019459989411000102 8290299

[CIT0003] Berlin, C.I., Hood, L.J., Wen, H., Szabo, P., Cecola, R.P., Rigby, P., & Jackson, D.F. (1993). Contralateral suppression of non-linear click-evoked otoacoustic emissions. *Hearing Research*, 71(1–2), 1–11. 10.1016/0378-5955(93)90015-S 8113128

[CIT0004] Campbell, D. (2013). Normative data. In F.R. Volkmar (Ed.), *Encyclopedia of autism spectrum disorders* (pp. 2062–2063). New York, NY: Springer. 10.1007/978-1-4419-1698-3_315

[CIT0005] Cassidy, J.W., & Ditty, K.M. (2001). Gender differences among newborns on a transient otoacoustic emissions test for hearing. *Journal of Music Therapy*, 38(1), 28–35. 10.1093/jmt/38.1.28 11407963

[CIT0006] Collet, L., Kemp, D.T., Veuillet, E., Duclaux, R., Moulin, A., & Morgon, A. (1990). Effect of contralateral auditory stimuli on active cochlear micro-mechanical properties in human subjects. *Hearing Research*, 43(2–3), 251–261. 10.1016/0378-5955(90)90232-E 2312416

[CIT0007] Collet, L., Veuillet, E., Bene, J., & Morgan, A. (1992). Effects of contralateral white noise on click-evoked emissions in normal and sensorineural ears: Towards an exploration of the medial olivocochlear system. *International Journal of Audiology*, 31(1), 1–7. 10.3109/00206099209072897 1554329

[CIT0008] De Ceulaer, G., Yperman, M., Daemers, K., Van Driessche, K., Somers, T., Offeciers, F.E., & Govaerts, P.J. (2001). Contralateral suppression of transient evoked otoacoustic emissions: Normative data for a clinical test set-up. *Otology & Neurotology*, 22(3), 350–355. 10.1097/00129492-200105000-00013 11347638

[CIT0009] Durante, A.S., & Carvallo, R.M.M. (2006). Changes in transient evoked otoacoustic emissions contralateral suppression in infants. *Pró-Fono Revista de Atualização Científica*, 18(1), 49–56. 10.1590/S0104-56872006000100007 16625871

[CIT0010] Durrheim, K. (2006). Basic quantitative analysis. In M. Terreblanche & K. Durrheim (Eds.), *Research in practice* (2nd ed., pp. 187–215). Cape Town: Juta.

[CIT0011] Edmonds, W. & Kennedy, T.D. (2017). *An applied guide to research designs : quantitative, qualitative, and mixed methods*. (2nd ed.). (pp. 117–119). SAGE Publications, Inc., Los Angeles. 10.4135/9781071802779

[CIT0012] Garinis, A.C., Glattke, T., & Cone-Wesson, B.K. (2008). TEOAE suppression in adults with learning disabilities. *International Journal of Audiology*, 47(10), 607–614. 10.1080/14992020802129402 18923982

[CIT0013] Geven, L.I., De Kleine, E., Free, R.H., & Van Dijk, P. (2011). Contralateral suppression of otoacoustic emissions in tinnitus patients. *Otology & Neurotology*, 32(2), 315–321. 10.1097/MAO.0b013e3181fcf180 20962699

[CIT0014] Guinan, J.J. (2006). Olivocochlear efferents: Anatomy, physiology, function, and the measurement of efferent effects in humans. *Ear and Hearing*, 27(6), 589–607. 10.1097/01.aud.0000240507.83072.e7 17086072

[CIT0015] Hall, J.W.I. (2017, September). *Rethinking your diagnostic audiology battery: using vale-added tests*. AudiologyOnline, Article 20463. Retrieved from https://www.audiologyonline.com/

[CIT0016] Hatzopoulos, S., Petrucell, J., Morkt, T., & Martini, A. (2003). TEOAE recording protocols revised: Data from adult subjects: Revision de protocolos de registro de TEOAE: Informacion de sujetos adultos. *International Journal of Audiology*, 42(6), 339–347. 10.3109/14992020309101327 14570242

[CIT0017] Hood, L.J., Berlin, C.I., Hurley, A., Cecola, R.P., & Bell, B. (1996a). Contralateral suppression of transient-evoked otoacoustic emissions in humans: Intensity effects. *Hearing Research*, 101(1–2), 113–118. 10.1016/S0378-5955(96)00138-4 8951438

[CIT0018] Hood, L.J., Berlin, C.I., Hurley, A., & Wen, H. (1996b). Suppression of otoacoustic emissions in normal hearing individuals. In C.I. Berlin (Ed.), *Hair cells and hearing aids* (pp. 57–72). Singular Press, San Diego.

[CIT0019] Joseph, J., Suman, A., Jayasree, G.K., & Prabhu, P. (2019). Evaluation of contralateral suppression of otoacoustic emissions in Bharatanatyam dancers and non-dancers. *The Journal of International Advanced Otology*, 15(1), 118–120. 10.5152/iao.2018.5645 30541728 PMC6483444

[CIT0020] Kalaiah, M.K., Nanchirakal, J.F., Kharmawphlang, L., & Noronah, S.C. (2017). Contralateral suppression of transient evoked otoacoustic emissions for various noise signals. *Hearing, Balance and Communication*, 15(2), 84–90. 10.1080/21695717.2017.1311504

[CIT0021] Kemp, D.T., Bray, P., Alexander, L., & Brown, A.M. (1986). Acoustic emission cochleography – Practical aspects. *Scandinavian Audiology, Supplementum*, 25, 71–95. Retrieved from http://europepmc.org/abstract/MED/3472324 3472324

[CIT0022] Kumar, P., Grover, V., Publius, A.S., Sanju, H.K., & Sinha, S. (2016). Assessment of rock musician’s efferent system functioning using contralateral suppression of otoacoustic emissions. *World Journal of Otorhinolaryngology – Head and Neck Surgery*, 2(4), 214–218. 10.1016/j.wjorl.2016.11.006 29204569 PMC5698541

[CIT0023] Kung, B.C., & Willcox, T.O., Jr. (2007). Examination of hearing and balance. In A.H.V. Schapira, E. Byrne, S. DiMauro, R.S.J. Frackowiak, R.T. Johnson, Y. Mizuno, … Z.K. Wszolek (Eds.), *Neurology and clinical neuroscience* (pp. 318–327). Philadelphia: Elsevier. 10.1016/B978-0-323-03354-1.50029-8

[CIT0024] McFadden, D., Pasanen, E.G., Raper, J., Lange, H.S., & Wallen, K. (2006). Sex differences in otoacoustic emissions measured in rhesus monkeys (Macaca mulatta). *Hormones and Behavior*, 50(2), 274–284. 10.1016/j.yhbeh.2006.03.012 16678823

[CIT0025] Mishra, S.K., & Lutman, M.E. (2013). Repeatability of click-evoked otoacoustic emission-based medial olivocochlear efferent assay. *Ear & Hearing*, 34(6), 789–798. 10.1097/AUD.0b013e3182944c04 23739244

[CIT0026] Muchnik, C., Ari-Even Roth, D., Othman-Jebara, R., Putter-Katz, H., Shabtai, E.L., & Hildesheimer, M. (2004). Reduced medial olivocochlear bundle system function in children with auditory processing disorders. *Audiology and Neurotology*, 9(2), 107–114. 10.1159/000076001 14981358

[CIT0027] Nobili, R., Vetešnik, A., Turicchia, L., & Mammano, F. (2003). Otoacoustic emissions from residual oscillations of the cochlear basilar membrane in a human ear model. *Journal of the Association for Research in Otolaryngology*, 4(4), 478–494. 10.1007/s10162-002-3055-1 14716508 PMC3202748

[CIT0028] Prasher, D., Ryan, S., & Luxon, L. (1994). Contralateral suppression of transiently evoked otoacoustic emissions and neuro-otology. *British Journal of Audiology*, 28(4–5), 247–254. 10.3109/03005369409086574 7735153

[CIT0029] Rasmussen, G.L. (1946). The olivary peduncle and other fiber projections of the superior olivary complex. *The Journal of Comparative Neurology*, 84(2), 141–219. 10.1002/cne.900840204 20982804

[CIT0030] Robinette, M.S., Cevette, M.J., & Probst, R. (2007). Otoacoustic emissions and audiometric outcomes across cochlear and retrocochlear pathology. In M.S. Robinette & T.J. Glattke (Eds.), *Otoacoustic emissions: Clinical applications* (3rd ed., pp. 236–237). Thieme Publishing, New York.

[CIT0031] Ryan, S., Kemp, D.T., & Hinchcliffe, R. (1991). The influence of contralateral acoustic stimulation on click-evoked otoacoustic emissions in humans. *British Journal of Audiology*, 25(6), 391–397. 10.3109/03005369109076614 1773199

[CIT0032] Sanches, S.G.G., & Carvallo, R.M. (2006). Contralateral suppression of transient evoked otoacoustic emissions in children with auditory processing disorder. *Audiology and Neurotology*, 11(6), 366–372. 10.1159/000095898 16988500

[CIT0033] Sarathy, K., & Jaya, V. (2017). Contralateral suppression of teoae in patients with tinnitus and normal hearing. *Biomedical Journal of Scientific & Technical Research*, 1(6), 1582–1584. 10.26717/BJSTR.2017.01.000492

[CIT0034] Siegel, J. (2008). Otoacoustic emissions. In R.H. Masland, T.D. Albright, P. Dallos, D. Oertel, S. Firestein, G.K. Beauchamp, … E.P. Gardner (Eds.), *The senses: A comprehensive reference* (pp. 237–261). Academic Press, New York.

[CIT0035] Stuart, A., & Cobb, K.M. (2015). Reliability of measures of transient evoked otoacoustic emissions with contralateral suppression. *Journal of Communication Disorders*, 58, 35–42. 10.1016/j.jcomdis.2015.09.003 26431768

[CIT0036] Urnau, D., & Tochetto, T.M. (2012). Occurrence and suppression effect of otoacoustic emissions in normal hearing adults with tinnitus and hyperacusis. *Brazilian Journal of Otorhinolaryngology*, 78(1), 87–94. 10.1590/S1808-86942012000100014 PMC9443871 22392244

[CIT0037] Van Zyl, A., Swanepoel, D., & Hall, J.W. (2009). Effect of prolonged contralateral acoustic stimulation on transient evoked otoacoustic emissions. *Hearing Research*, 254(1), 77–81. 10.1016/j.heares.2009.04.013 19401226

[CIT0038] Vasconcelos, R.M., Serra, L.S.M., & De Farias Aragão, V.M. (2008). Emissões otoacústicas evocadas transientes e por produto de distorção em escolares. *Revista Brasileira De Otorrinolaringologia*, 74(4), 503–507. 10.1590/S0034-7299200800040000418852974

[CIT0039] Velenovsky, D.S., & Glattke, T.J. (2002). The effect of noise bandwidth on the contralateral suppression of transient evoked otoacoustic emissions. *Hearing Research*, 164(1–2), 39–48. 10.1016/S0378-5955(01)00393-8 11950523

[CIT0040] Veuillet, E., Collet, L., & Duclaux, R. (1991). Effect of contralateral acoustic stimulation on active cochlear micromechanical properties in human subjects: Dependence on stimulus variables. *Journal of Neurophysiology*, 65(3), 724–735. 10.1152/jn.1991.65.3.724 2051201

[CIT0041] Von Specht, H., Ganz, M., Pethe, J., Leuschner, S., & Pytel, J. (2001). Linear versus non-linear recordings of transiently-evoked otoacoustic emissions – Methodological considerations. *Scandinavian Audiology*, 30(1), 116–118. 10.1080/010503901300007263 11318439

